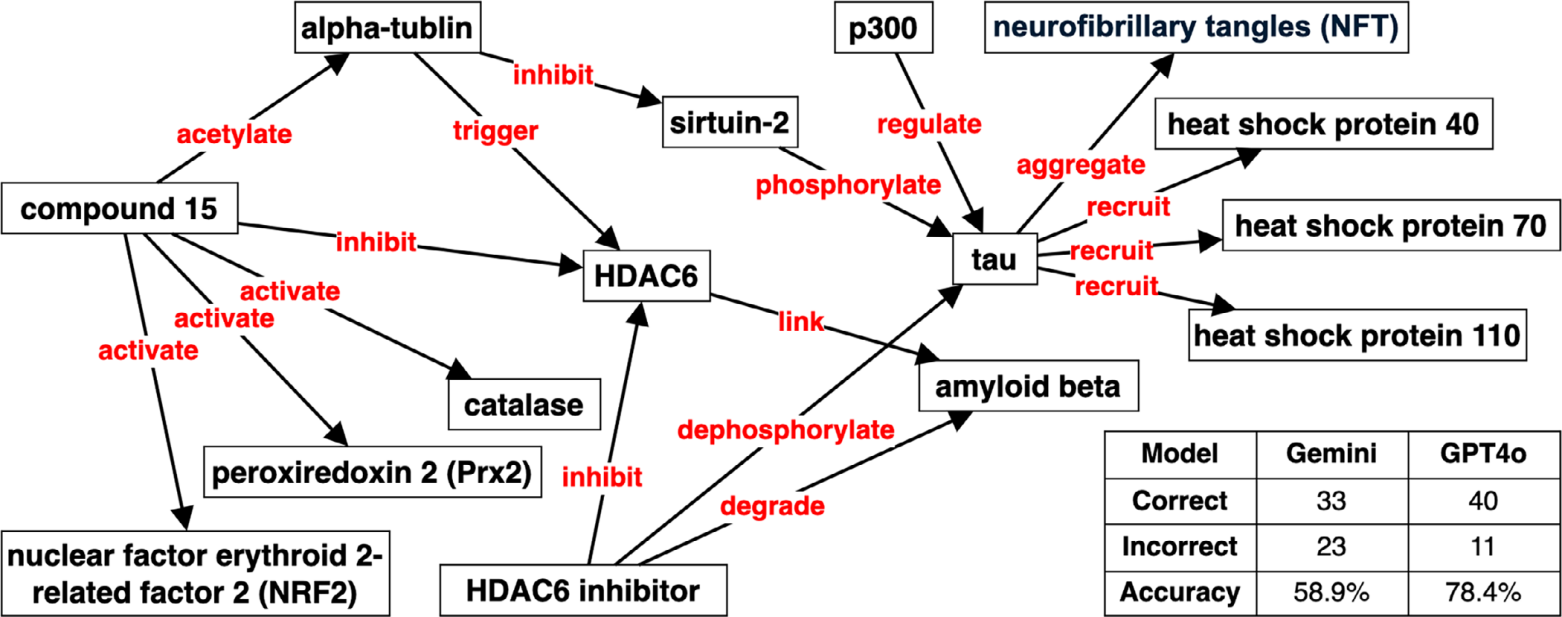# Mapping Molecular Pathways of Histone Deacetylase in Alzheimer's Disease with Large Language Model‐Driven Knowledge Discovery

**DOI:** 10.1002/alz70855_104141

**Published:** 2025-12-23

**Authors:** Aneesh Mazumder, Anirudh Mazumder, Claire Suen, Prasenjit Mondal, Can Zhang, Haoqi Sun

**Affiliations:** ^1^ Harvard College, Cambridge, MA, USA; ^2^ Texas Academy of Mathematics and Science, University of North Texas, Denton, TX, USA; ^3^ Massachusetts General Hospital, Charlestown, MA, USA; ^4^ Beth Israel Deaconess Medical Center, Boston, MA, USA

## Abstract

**Background:**

Despite the classical pathologies in Alzheimer's Disease (AD), novel molecular pathways such as histone deacetylase 6 (HDAC6) have shown promising results. With the growing literature, we need systematic approaches to study the intricate molecular pathways. The revolution in large language models (LLMs) presents an innovative and powerful approach to summarizing and discovering the vast knowledge in the whole field by automatically reading and understanding extensive literature. The results can be represented by knowledge graphs (KGs) that map molecular interactions and pathways in AD.

**Method:**

The corpus contained 265 papers from PubMed using the keywords “(Alzheimer's disease OR AD) AND (histone deacetylase OR HDAC)” in abstract/title and in human studies, on Sep 7, 2024. Here, we used the abstract part of the paper only. The preprocessing involved abbreviation expansion and coreference parsing. We used two LLMs: GPT‐4o and Gemini. The LLMs processed each sentence individually to extract subject‐predicate‐object triplets using finetuned prompts, where the subject/object must be molecules. To standardize the verbs, we prompted LLMs to map the verb to one of 34 predefined verb categories. To standardize the subject/object, we queried the UniProt database and used LLM to find the best candidate standardized name. The triplets from multiple papers were combined to form a KG. Performance was evaluated based on human‐assessed accuracy of triplets from 10 randomly paper abstracts.

**Result:**

Figure 1 shows the resulting KG from the 10 paper abstracts. It contained 15 molecules as the nodes and 17 interactions as the arrows. Tau, compound 15, and HDAC6 were the hub nodes. The table in Figure 1 in the lower right corner shows that GPT‐4o outperformed Gemini, achieving an accuracy of 78.4%, compared to Gemini's 58.9%.

**Conclusion:**

Leveraging LLMs such as GPT‐4o, we efficiently extracted structured knowledge from the field of AD and HDAC. The KG represents a foundational step to systematically understand what's known and what are the major gaps for novel therapeutic targets. The approach holds high potential even for broader scientific fields and automation of science.